# Potential role of the ABCG2-Q141K polymorphism in type 2 diabetes

**DOI:** 10.1371/journal.pone.0260957

**Published:** 2021-12-02

**Authors:** Edit Szabó, Anna Kulin, Orsolya Mózner, László Korányi, Botond Literáti-Nagy, Márta Vitai, Judit Cserepes, Balázs Sarkadi, György Várady

**Affiliations:** 1 Institute of Enzymology, ELKH Research Centre for Natural Sciences, Center of Excellence by Hungarian Academy of Sciences, Budapest, Hungary; 2 Doctoral School of Molecular Medicine, Semmelweis University, Budapest, Hungary; 3 Drug Research Center, Balatonfüred, Hungary; 4 Research Institute of Biomolecular and Chemical Engineering, University of Pannonia, Veszprém, Hungary; 5 CellPharma Kft, Budapest, Hungary; 6 Department of Biophysics and Radiation Biology, Semmelweis University, Budapest, Hungary; Indiana University Purdue University at Indianapolis, UNITED STATES

## Abstract

Type 2 diabetes mellitus (T2DM) is a complex metabolic disease and variations in multispecific membrane transporter functions may affect T2DM development, complications or treatment. In this work we have analyzed the potential effects of a major polymorphism, the Q141K variant of the ABCG2 transporter in T2DM. The ABCG2 protein is a multispecific xeno- and endobiotic transporter, affecting drug metabolism and playing a key role in uric acid extrusion. The ABCG2-Q141K variant, with reduced expression level and function, is present in 15–35% of individuals, depending on the genetic background of the population, and has been shown to significantly affect gout development. Several other diseases, including hypertension, chronic renal failure, and T2DM have also been reported to be associated with high serum uric acid levels, suggesting that ABCG2 may also play a role in these conditions. In this work we have compared relatively small cohorts (n = 203) of T2DM patients (n = 99) and healthy (n = 104) individuals regarding the major laboratory indicators of T2DM and determined the presence of the SNP *rs2231142* (*C421A*), resulting the ABCG2-Q141K protein variant. We found significantly higher blood glucose and HbA1c levels in the T2DM patients carrying the ABCG2-Q141K variant. These findings may emphasize the potential metabolic role of ABCG2 in T2DM and indicate that further research should explore how prevention and treatment of this disease may be affected by the frequent polymorphism of *ABCG2*.

## Introduction

Type 2 diabetes mellitus (T2DM) is one of the most common metabolic disorders, associated with elevated blood glucose levels due to insulin resistance and/or relative insulin deficiency. T2DM, comprising of about 90% of all diabetes cases, is a multifactorial disease, thus genetic background, lifestyle, hormonal changes, infections or medications may all play a role in disease development, making difficult to establish accurate diagnosis and therapy. If T2DM persists, this condition greatly increases the risk of heart disease, stroke, kidney failure, lower limb ischemia, and retinopathy [1, 2].

ABCG2 (BCRP, MXR) is a homodimeric ABC transporter whose main function is the extrusion of harmful compounds, the transport of xeno- and endobiotics across the plasma membrane and tissue barriers, thus being a key player in the so called “chemoimmunity” [3]. This protein is expressed in numerous tissues, including the intestine, blood-brain barrier, placenta and various stem cells [4]. ABCG2 plays an important role in modulating the absorption, distribution, metabolism, excretion and toxicity (ADME-Tox) properties of various drugs, and is also involved in the multidrug resistance of cancer cells [5, 6].

ABCG2 is responsible for the extrarenal (mainly intestinal) extrusion of uric acid, being one of the key uric acid transporters in the enterocytes, while this transporter is also present and functional in uric acid export in the kidney proximal tubules [7, 8]. Genome-wide association (GWA) studies have shown that decreased expression level and/or function of ABCG2 are associated with elevated serum uric acid levels, an important risk factor for gout development [9]. Since several disease conditions, including hypertension, chronic renal failure, and T2DM are also reported to be associated with high serum uric acid levels, ABCG2 may also play a role in these cases [10, 11].

Genetic studies revealed 40–70% heritability for serum uric acid levels [12], while in studies investigating the relationship between high serum uric acid and T2DM, the association between blood glucose and serum uric acid levels was inconsistent [10]. This variability may be attributed to inherited variations in the expression and function of both uric acid and hexose transporters, while high serum uric acid was found to be present in 20–30% of the T2DM patients [10, 11]. In addition, complications of T2DM, e.g. diabetic nephropathy, retinopathy, and cardiovascular disease were found to be closely related to high serum uric acid levels [10–13].

GWA and directed molecular genetic studies have identified decreased ABCG2 membrane expression levels associated with the relatively common polymorphism (*rs2231142*), resulting in the ABCG2-Q141K protein variant [13–16]. This variant occurs in about 15–20% of people of European descent (minor allele frequency values (MAF) 0.102605), while its frequency is much higher in Asian populations (about 30–35%, MAF values 0.2967) [17]. Folding and cellular processing of this ABCG2 variant is impaired; thus, plasma membrane expression is reduced [12, 16]. It has been shown that the *ABCG2 C421A* polymorphism contributes to the development of high uric acid levels and may alter the pharmacokinetics of ABCG2 substrate drugs. As an example, this polymorphism was shown to significantly influence the pharmacokinetics of fluvastatin and simvastatin lactone, but not of pravastatin or simvastatin acid [18].

In the present work, we have compared cohorts of T2DM patients and healthy individuals in terms of HbA1c (hemoglobin A1c), blood glucose and uric acid levels, and determined the presence of the SNP leading to the ABCG2 protein variant (ABCG2-Q141K). We found significant correlations between the metabolic parameters and the ABCG2 polymorphism among diabetic patients.

## Methods

### Description of the patient and control populations—Key data

The samples from T2DM patients were obtained from the Drug Research Centre (DRC, Balatonfüred, Hungary). The key laboratory diagnostic parameters analyzed here for T2DM patients and the healthy volunteers included HbA1c, glucose, and uric acid levels (Table 1).

**Table 1:** Summary of some key data for age-matched control subjects and the T2DM patients.

	n	HbA1c	HbA1c	glucose	UA
		mmol/mol	%	mM	μM
CTRL	104	38±1	5.6±0.1	5.4±0.2	314.9±8.7
T2DM	99	49±1	6.7±0.1	7.1±0.4	334.0±8.9
p		**<0.0001**	**<0.0001**	**<0.0001**	0.1260

The values are expressed as means ± SE. The p values were calculated by Student’s t-test.

The age-matched healthy volunteers were selected from visitors at the clinic not suffering from diabetes or related metabolic diseases. The average age ± SD in the control group was 65±12 years, in the group of T2DM patients it was 70±12 years. In this study was examined 87 male and 116 female. A group of the T2DM patients (n = 36) were first time visitors at the DRC diabetic clinic, showing insulin resistance. The long-time successfully managed (treated) T2DM patients (n = 63) had dietary supervision and obtained metformin as a key treatment. This treatment was supplemented in some cases with oral antidiabetics and also with allopurinol (milurit), to reduce uric acid levels. More detailed laboratory and treatment data for the patients examined here are available in ref [19].

The clinical diagnosis of T2DM was established according to the criteria of the American Diabetes Association (ADA) [20]. The study was approved by the Scientific and Research Committee of the Medical Research Council, Hungary (ETT TUKEB references: 19680-3/2019/EKU, 2367-1/2019/EKU). All methods were performed in accordance with the relevant guidelines and regulations. All control subjects and patients in the study gave informed consent to participate in this research.

### SNP analysis for ABCG2

Genomic DNA was purified from 300 μL of EDTA-anticoagulated blood samples with Puregene Blood Kit (Qiagen). TaqMan-based qPCR reactions for *ABCG2-C421A* (*rs2231142*) SNP (cat. 436269, C__15854163_70) detection were performed in a StepOnePlus device (Applied Biosystems) with premade assay mixes and a master mix (cat. 4371353) from Thermo Fisher. TaqMan probe specificity was verified by sequencing.

#### Statistics

The laboratory data values are expressed as means ± standard error. The differences of the key data for age-matched control subjects and the T2DM patients were analyzed by Student’s t-test (GraphPad Prism 8.0.1). The number of patients (n) involved in each analysis is indicated in the respective tables.

## Results

### 1. Genetic SNP analysis

We have examined the frequency of the SNP *rs2231142*, also known as *C421A*, leading to the ABCG2-Q141K polymorphism among the control and the T2DM patients. Although the MAF values and the chi-square values, representing the compliance with a Hardy-Weinberg distribution (HW) were somewhat different between the patient and the control groups, this difference was not statistically significant (Table 2).

**Table 2:** The occurrence of the SNP *rs2231142* (ABCG2-Q141K variant) in the healthy and T2DM populations.

	total n	homozygous	heterozygous	wild type	MAF	HW (Chi^2^)
CTRL	104	2	25	77	0.14	1
T2DM	99	0	23	76	0.116	0.47
all	203	2	48	153	0.13	0.81

MAF (minor allele frequency), and HW (Hardy-Weinberg) equilibrium values were calculated from the healthy, age-matched control population, T2DM patients or merge of the two groups.

### 2. Laboratory data analysis

**Fig 1:**
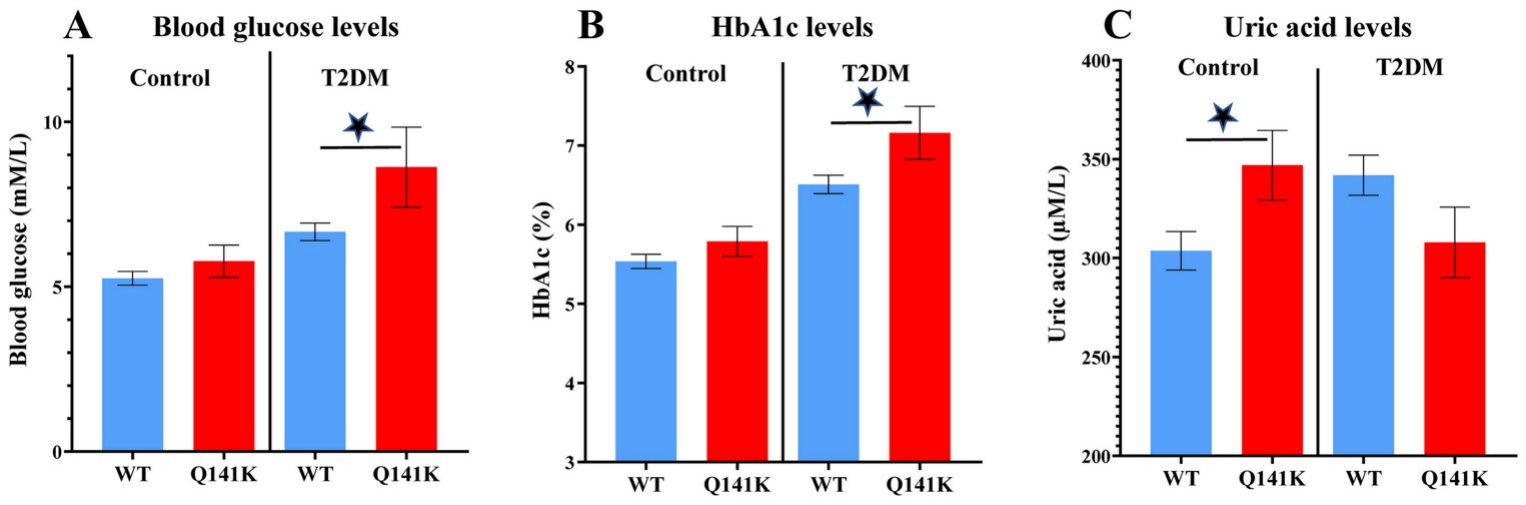
Blood glucose (Panel A), HbA1c (Panel B) or uric acid (Panel C) levels and the presence of the Q141K variant (both hetero- and homozygotes) in the groups of healthy individuals and T2DM patients. Values are expressed as means ± SE. Blue: ABCG2-wild type, red: ABCG2-Q141K. Star (*) indicates a significant difference obtained in the individuals carrying the Q141K polymorphism *p<0.05. The p values were calculated by Student’s t-test.

A basic laboratory measurement reflecting glucose metabolism and orienting towards the presence of T2DM is fasting blood glucose level. In the following we compared venous blood glucose values, as measured in healthy and T2DM patients, respectively, and the presence of the SNP *rs2231142* in these individuals (Fig 1A, Table 3A and S2 Table).

**Table 3:** Statistical analysis of the correlation of blood glucose (Panel A), HbA1c (Panel B) or uric acid (Panel C) levels and the presence of the Q141K variant in the groups of healthy individuals and T2DM patients.

		Significance of differences	healthy Q141K	T2DM Q141K	healthy WT
**A**	**blood glucose**	healthy WT	0.2518	**<0.0001**	-
		T2DM WT	0.0968	***0*.*0188***	**<0.0001**
		T2DM Q141K	**0.0255**	-	-
**B**	**HbA1c**	healthy WT	0.1834	**<0.0001**	-
		T2DM WT	**0.0019**	***0*.*0228***	**<0.0001**
		T2DM Q141K	**0.0006**	-	-
**C**	**uric acid**	healthy WT	**0.0287**	0.8348	-
		T2DM WT	0.8041	*0*.*1072*	**0.0073**
		T2DM Q141K	0.129	-	-

The p values were calculated by Student’s t-test.

In general, as expected, average blood glucose levels in the T2DM patients were significantly higher than in the healthy controls (see Table 3A). In the healthy volunteers, the presence of the ABCG2-Q141K variant did not significantly affect the measured blood glucose levels. However, in the T2DM patients carrying the SNP, blood glucose levels were significantly greater than in patients carrying the wild type alleles. Although the number of patients in these groups was relatively small, we have also analyzed these differences between the first-time visitors at the clinic (showing early signs of T2DM, eg. glucose intolerance) and the successfully, long-time treated patients (S1A Fig). In the latter group the presence of the ABCG2-Q141K polymorphism correlated with a significantly increased fasting glucose level, while in the case of the first-time visitors there was no such difference.

Hemoglobin A1c levels are the best available indicators for a long-term increase in blood glucose levels which significantly affect the occurrence of disease-related complications in T2DM patients. In the following analysis, we compared HbA1c levels between the groups of control individuals and T2DM patients, respectively, considering the presence of the *ABCG2- C421A* SNP in these groups.

As shown in Fig 1B and Table 3B, HbA1c levels were significantly higher in the T2DM patients, as compared to the control subjects, regardless of the presence or absence of the *ABCG2-Q141K* SNP. There was no significant difference in the HbA1c levels measured in the healthy control individuals, with or without the presence of this SNP. However, HbA1c values were significantly higher in T2DM patients carrying the *ABCG2- C421A* SNP, when compared to patients carrying only the wild type alleles. As shown in S1B Fig, this correlation of the HbA1c levels with the SNP was not significant in the first-time visitors (untreated) at the diabetes clinic, while the increased HbA1C levels showed a significant correlation with the *ABCG2-C421A* SNP in the long-time treated patients. Thus, a successful management of the T2DM patients did not compensate for the adverse metabolic effect of the ABCG2-Q141K polymorphism.

Uric acid levels have been found to be affected by the presence of the *ABCG2- C421A* SNP in several studies [21]. Indeed, when examining the uric acid levels in the control subjects, we found significantly higher uric acid levels in individuals carrying this SNP (Fig 1C and Table 3C). In general, uric acid levels in the T2DM patient group also appeared to be greater than these levels in the control individuals, although this difference was not significant (see Table 1). However, in the T2DM patients carrying the *ABCG2- C421A* SNP, the mean uric acid levels were not statistically significant from those in the patients carrying the wild type allele.

## Discussion

In the present study we have examined the potential relationship between the presence of the ABCG2-Q141K protein variant and the basic metabolic parameters in T2DM patients, as compared to a group of healthy individuals. We focused on the potential correlations between the occurrence of this SNP variant and the blood glucose, HbA1c, and uric acid levels.

The *ABCG2*-Q141K variant is a frequent polymorphism in the Caucasian and Asian populations. Both in vitro and in vivo studies documented a functional impairment of this variant, caused by reduced stability and lower plasma membrane expression of the ABCG2-Q141K transporter protein (S2 Fig) [22, 23]. Several reports indicate that this variant significantly contributes to elevated serum uric acid levels and gout development [9, 21, 24], and complications affecting T2DM patients may also be related to increased serum uric acid levels [10, 12]. According to the T2D Knowledge Portal (https://t2d.hugeamp.org/), summarizing the T2DM GWA studies [25], although at a relatively low level, a significant association was found between increased HbA1c levels and *ABCG2*-Q141K polymorphisms, while no significant correlation was observed between fasting blood glucose levels and this polymorphism (see details in S3 Fig, S1 Table). Since ABCG2 function is also an important determinant of the ADME-tox properties of numerous drugs, including those (e.g. metformin, glyburide, or certain statins) used in the treatment of T2DM, this polymorphism may also affect the treatment efficacy of T2DM [26–30].

In the present study, while including only relatively small cohorts, we found that T2DM patients carrying the *ABCG2-C421A* SNP had significantly higher blood glucose levels than patients carrying only the wild-type alleles. In addition, the HbA1c levels, reflecting long-term blood glucose alterations, were also significantly higher in the T2DM patients carrying this SNP. In contrast, in healthy, control individuals, there was no such difference in the blood glucose or HbA1c levels, correlating with the presence of the ABCG2-Q141K polymorphism. These data suggest that the presence of the functionally impaired ABCG2-Q141K variant, even in properly managed patients, corresponds to an impaired treatment effect of T2DM patients, regarding both their short-term and long-term blood glucose levels. Thus, the presence of this ABCG2 variant may promote the occurrence of disease complications related to long-term high blood glucose levels.

When analyzing the levels of plasma uric acid, we found, as expected, that these levels were significantly increased in the control populations by the presence of the ABCG*2*-Q141K polymorphism. However, this polymorphism did not correlate with increased uric acid levels in the type 2 diabetic patients. This may reflect the drug treatment protocols of these patients, which also included the xanthine oxidase inhibitor allopurinol in several cases.

As a summary, our current data indicate that the presence of the frequent ABCG2-Q141K variant has an unfavorable effect on the blood glucose and HbA1c levels even in the long-time properly managed T2DM patients. The higher fasting blood glucose and HbA1c levels found in this patient group may indicate a combined effect of the ABCG2-Q141K polymorphism on the long-term glucose handling, and possibly on antidiabetic drug metabolism in these patients. Although the present work is based on a relatively small number of patients, this is a first direct demonstration of this association and a further detailed investigation of these findings may help to clarify the complex role of this ABCG2 variant in T2DM. Still, our current data strongly suggest that the management of the subgroup of T2DM patients carrying the ABCG2-Q141K variant should be carefully followed and may require a specific treatment protocol.

## Supporting information

S1 Fig Blood glucose (A) and HbA1c (B) levels and the presence of the Q141K variant in the groups of healthy individuals, untreated (first time visitors at the diabetes clinic) and clinically managed (treated) T2DM patients. Values are expressed as means ± SE. Blue: ABCG2-wild type, red: ABCG2-Q141K. Star (*) indicates a significant difference obtained in the individuals carrying the Q141K polymorphism *p<0.05. The p values were calculated by Student’s t-test. The n values: control individuals (104): WT = 77, Q141K = 27; first time visitors, untreated (36): WT = 29, Q141K = 7; successfully managed, treated (63): WT = 47, Q141K = 16.
(TIF)

S2 Fig ABCG2 expression levels and the presence of the Q141K variant (both hetero- and homozygotes) in the groups of healthy individuals and T2DM patients. We have measured the expression levels of ABCG2 in the RBC membranes of normal healthy individuals and T2DM patients. The methods see in ref [19]. In brief, the fixed and permeabilized RBC membranes (ghosts) were incubated with the Bxp34 monoclonal primer antibody (Abcam, cat. ab3379) followed by a secondary Alexa Fluor 488-labeled goat anti-mouse (H + L) antibody (Thermo Fisher, A-11001), in 96 well plates. RBC ghosts were analyzed for antibody staining by Attune NxT acoustic flow cytometer. The differences between the protein expression values of the groups were analyzed by Mann-Whitney U-test (GraphPad Prism 8.0.1).
(TIF)

S3 Fig The forest plot shows the phenome-wide association of ABCG2-Q141K for uric acid, HbA1c and fasting glucose traits. The PheWAS associations were generated by bottom-line meta-analysis across all datasets in the Type 2 Diabetes Knowledge Portal (https://t2d.hugeamp.org). The color of the cubes shows the p values (red: p ≤5.00e-8, pink: 5.00e-8 < p ≤ 2.50e-6, gray: p > 0.05). The gray line represents the 95% confidence interval.
(TIF)

S1 Table The details of the PheWAS associations of ABCG2-Q141K for uric acid, HbA1c and fasting glucose traits (from https://t2d.hugeamp.org). **A**, Summary of the PheWAS data. **B**, Key data from the databases used in the meta-analysis. The p values were calculated by bottom-line meta-analysis across all datasets in the Type 2 Diabetes Knowledge Portal. The Beta coefficient is the estimated difference in a phenotype between a heterozygous carrier of the effect allele and a homozygous reference allele carrier. Green arrow: positive correlation between the effect allele and the phenotype, red arrow: negative correlation.
(TIF)

S2 Table The datasets analyzed during the current study. (TIF)
