# Supplementary figures and images for: Potential role of the ABCG2-Q141K polymorphism in type 2 diabetes
Source: PLoS One. 2021 Dec 2;16(12):e0260957. doi: 10.1371/journal.pone.0260957 (PMC8638943; doi:10.1371/journal.pone.0260957)

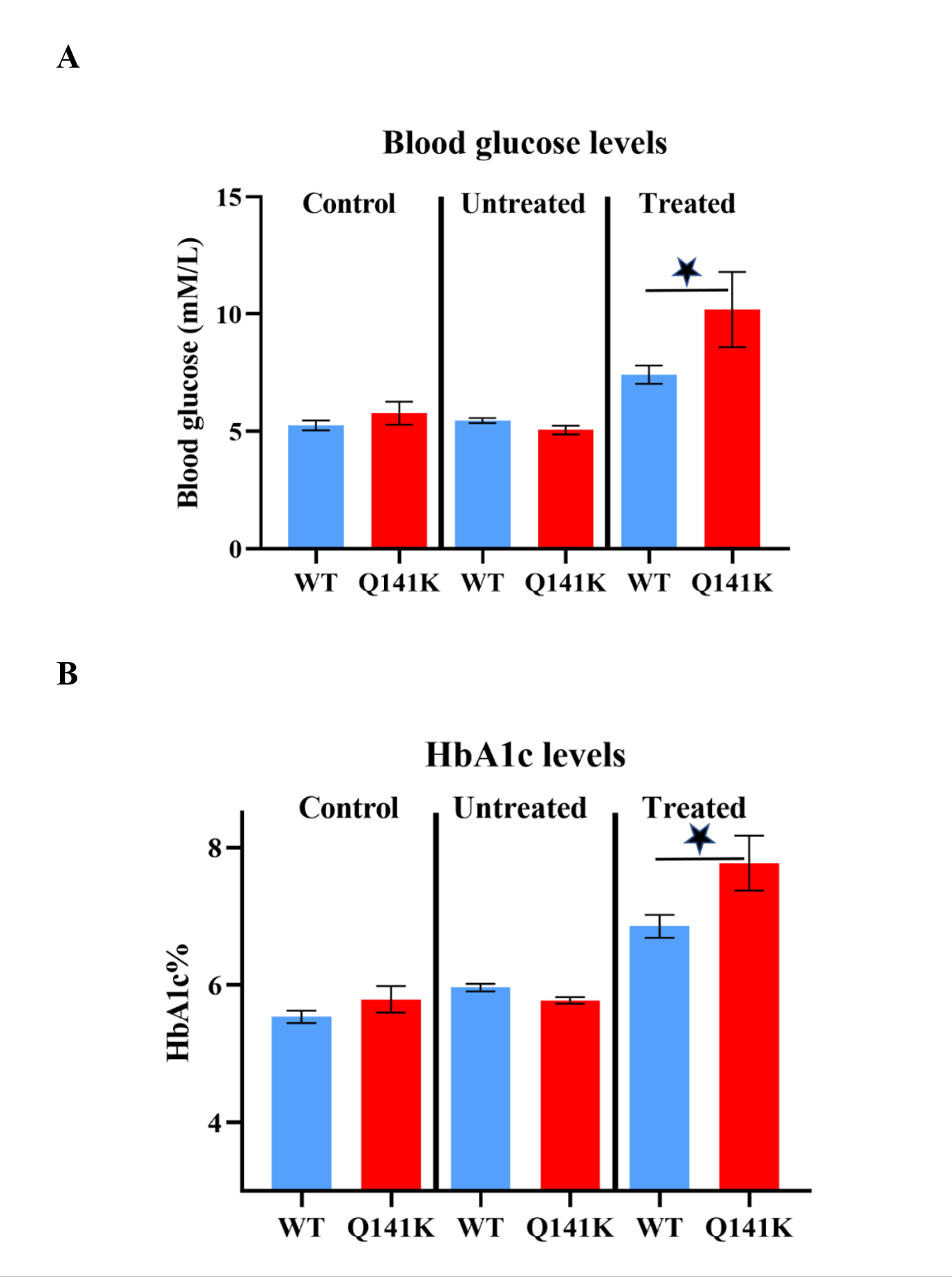

Supplement: S1 Fig — Values are expressed as means ± SE. Blue: ABCG2-wild type, red: ABCG2-Q141K. Star (*) indicates a significant difference obtained in the individuals carrying the Q141K polymorphism *p<0.05. The p values were calculated by Student’s t-test. The n values: control individuals (104): WT = 77, Q141K = 27; first time visitors, untreated (36): WT = 29, Q141K = 7; successfully managed, treated (63): WT = 47, Q141K = 16. (TIF) [file pone.0260957.s001.tif]

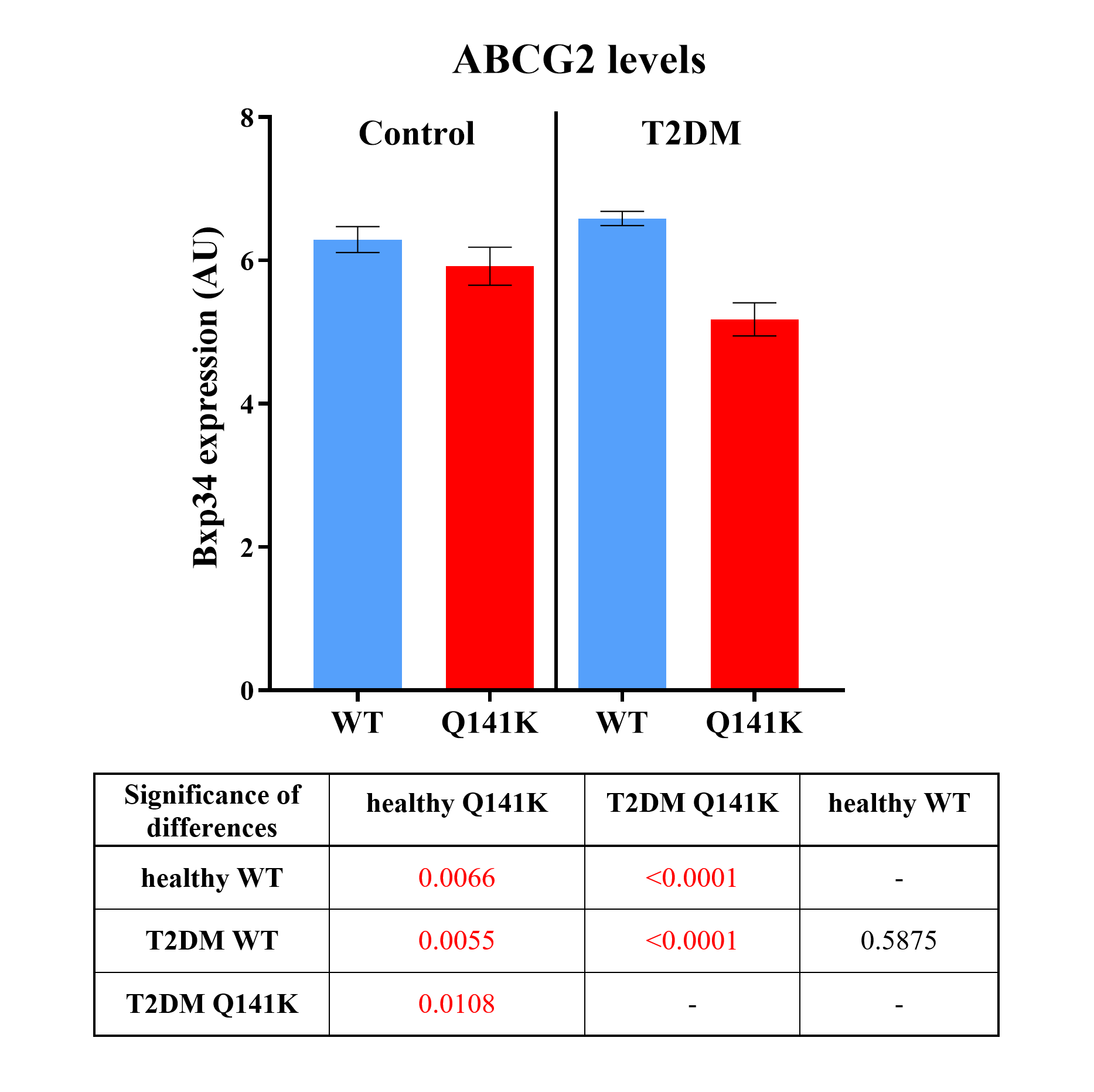

Supplement: S2 Fig — We have measured the expression levels of ABCG2 in the RBC membranes of normal healthy individuals and T2DM patients. The methods see in ref [19]. In brief, the fixed and permeabilized RBC membranes (ghosts) were incubated with the Bxp34 monoclonal primer antibody (Abcam, cat. ab3379) followed by a secondary Alexa Fluor 488-labeled goat anti-mouse (H + L) antibody (Thermo Fisher, A-11001), in 96 well plates. RBC ghosts were analyzed for antibody staining by Attune NxT acoustic flow cytometer. The differences between the protein expression values of the groups were analyzed by Mann-Whitney U-test (GraphPad Prism 8.0.1). (TIF) [file pone.0260957.s002.tif]

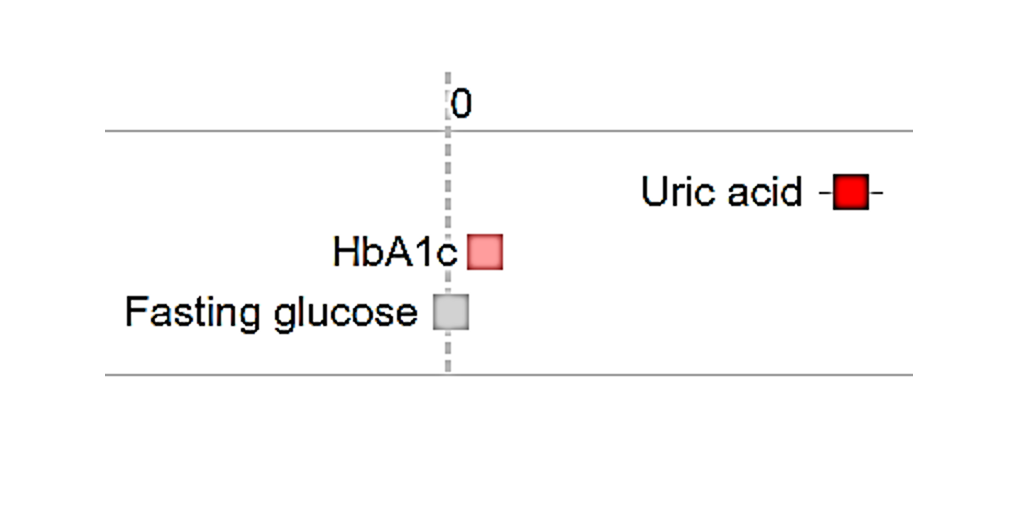

Supplement: S3 Fig — The PheWAS associations were generated by bottom-line meta-analysis across all datasets in the Type 2 Diabetes Knowledge Portal (https://t2d.hugeamp.org). The color of the cubes shows the p values (red: p ≤5.00e-8, pink: 5.00e-8 < p ≤ 2.50e-6, gray: p > 0.05). The gray line represents the 95% confidence interval. (TIF) [file pone.0260957.s003.tif]

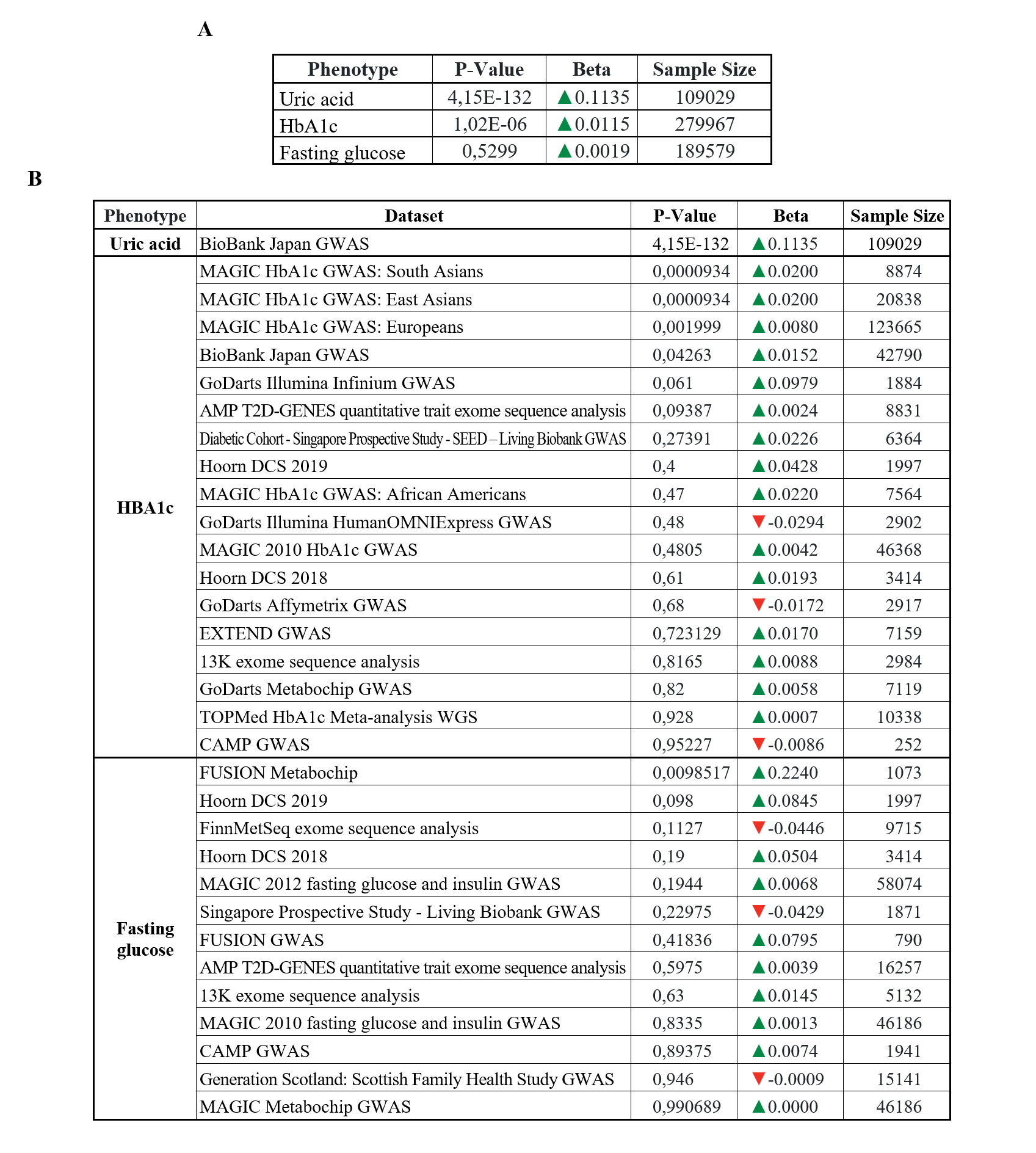

Supplement: S1 Table — A, Summary of the PheWAS data. B, Key data from the databases used in the meta-analysis. The p values were calculated by bottom-line meta-analysis across all datasets in the Type 2 Diabetes Knowledge Portal. The Beta coefficient is the estimated difference in a phenotype between a heterozygous carrier of the effect allele and a homozygous reference allele carrier. Green arrow: positive correlation between the effect allele and the phenotype, red arrow: negative correlation. (TIF) [file pone.0260957.s004.tif]

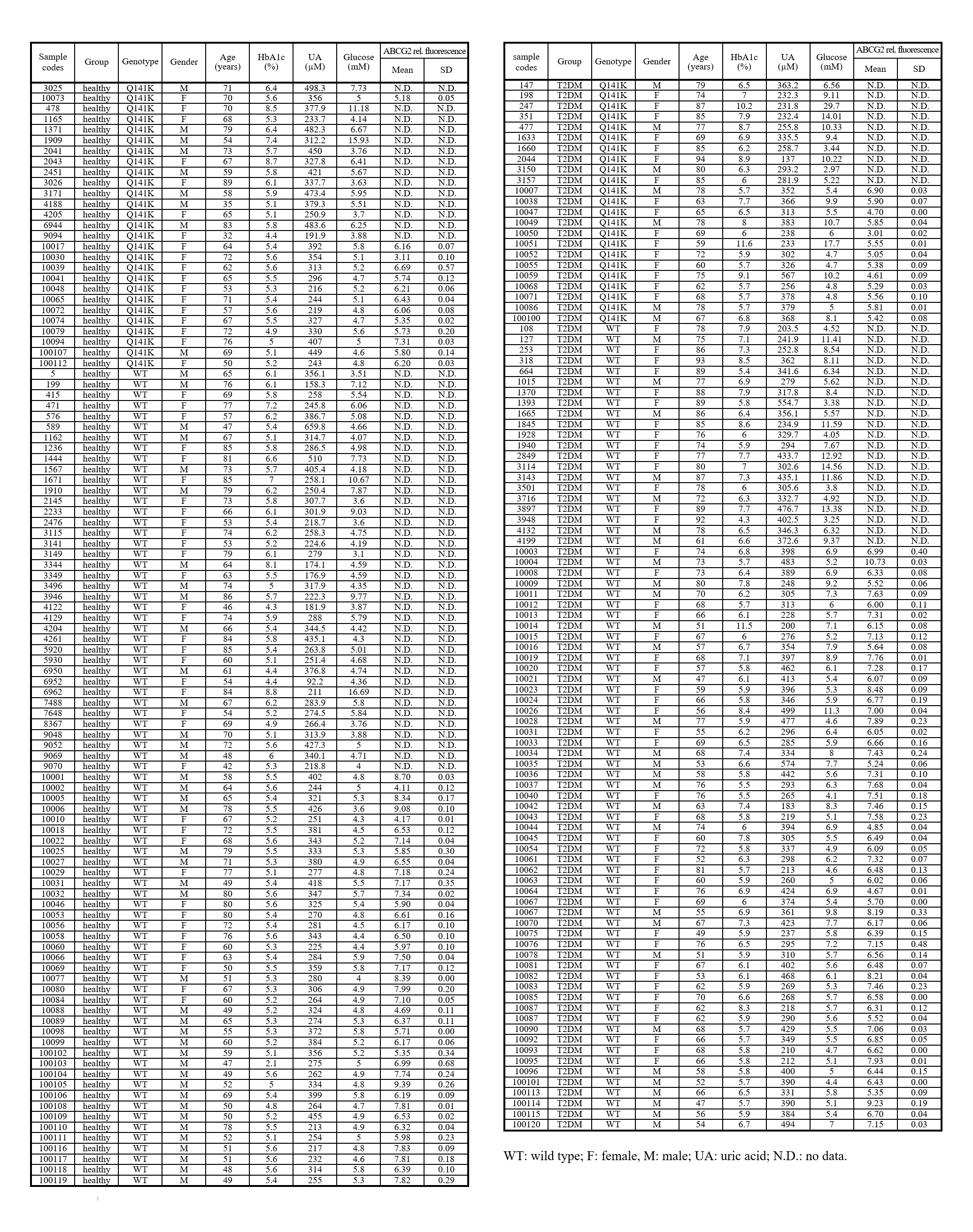

Supplement: S2 Table — (TIF) [file pone.0260957.s005.tif]
